# Address to Marquis Cornwallis, on Vaccine Inoculation

**Published:** 1802-03

**Authors:** G. C. Jenner

**Affiliations:** Burbage, Wilts


### *To the Editors of the Medical and Phyfical Journal.*

GENTLEMEN,

HAVING read in your Medical and Phyfical Journal, fome Queries refpecting the *imperfect* or *fpurious* Vaccine puftule, together with fome obfervations on the fuppofed caufe of the occafional deviation from the genuine character of the difeafe, I beg leave to offer a few remarks from the refult of my own experience, with an ardent wifh that they may throw a ray of light on a fubject which fo intimately concerns the deareft interefts of humanity.

At the commencement of my practice in this branch of the Medical Profeffion, I generally took the Vaccine Virus for inoculation after the puftule was arrived at maturity, and the *areola* completely formed, becaufe I then found the fluid in the greateft quantity; and as I had frequently from one to two hundred perfons of a day apply to be inoculated, the *quantity* of virus became a material object to me. The confequence was, that in fome inftances the operation failed of producing any, and in others but an imperfect effect. I was the more mifled by obferving the Vaccine matter to retain its entire fluidity till a late ftage of the difeafe, even (as fubfequent experiments have convinced me) after its active or fpecific properties were entirely loft. When I had a fufficient number of patients to furnifh me with *early* virus, I inoculated upwards of 200 perfons with it *in a recent fluid ftate*, and had the fatisfaction to find that they were all infected, and went through the complaint in the moft perfect and fatisfactory manner. I was now induced to believe that ufing *late* virus was the principal, if not the fole caufe of the fpurious puftule, and accordingly made it a rule rule *never to inoculate from an arm after the eighth or ninth day, or when the efflorefcence furrounding the puftule was formed to any confiderable extent*; and by a rigid obfervance of this law, I have feldom failed of producing the perfect Vaccine from the firft inoculation: But as fome few inftances of a fpurious puftule have occurred in my practice, notwithftanding the above precautions, we muft not regard the ufe of late virus as the *only* inefficient caufe, although I have reafon to believe it to be the moft frequent one.

An imperfect puftule now and then arifes from the ufe of virus previoufly dried too haftily by the fire. The heat, if it does not deftroy the action of the Vaccine fluid altogether, frequently decompofes it, and renders its prefervative powers doubtful; for it is on the complete puftule alone we are to depend for fecurity againft a future attack of the Small-pox.

In two inftances I have feen the *perfect* and *fpurious* puftule co-exiftent on the fame perfon, for which I do not prefume to affign a caufe. There are, no doubt, as in the Small-pox, if we may reafon from analogy, many circumftances that may confpire to fufpend the action of the Vaccine virus, or render its effect incomplete. Thefe occurrences, however, are rare; but whenever they do appear, I would recommend a re-inoculation of the patient. But from whatever fource the fpurious puftule may arife, there is this fatisfaction, that it is very eafily diftinguifhed from the perfect difeafe by thofe who have paid any attention to the Vaccine practice. The features of the genuine difeafe are ftrongly marked, and require but little difcernment to be familiarly acquainted with them.

Perfons who have previoufly had the Small-pox are fometimes fufceptible of the local action of the Vaccine virus, and a fpurious puftule is produced, altho' it oftener fails of exciting any effect whatever. I have feen a few inftances where the perfect difeafe has been produced under fimilar circumftances, one of which I fhall mention. I repeatedly inoculated myfelf with Vaccine matter, but never could excite the leaft inflammation till I had repeated the operation fifty times. The fiftieth inoculation produced a fpurious puftule, which was premature in its progrefs, and terminated the eighth day in a brown polifhed fcab. A drawing of this puftule was taken by an ingenious artift (Mr. William Cuff) and is now in the poffeffion of my uncle, Dr. Jenner. Two cafes of perfect puftule after the Small-pox have come under my obfervation: One was a boy, inoculated by Mr. Fofter, Surgeon, of Thornbury, in Gloucefterfhire. The other happened to myfelf from the following circumftance. A young lady of a timid difpofition, dreading the operation of inoculation, wifhed to be fhewn in what manner ner it was performed. I ſhewed her by making a ſlight puncture on the back of my hand with a lancet, on the point of which was a little dried Vaccine virus. About the fifth day I felt an itching, and obſerved a ſlight veſication, which I readily perceived to be the Vaccine. This increaſed in ſize till the ninth day, when it was ſurrounded by an efflorefence, and then declined in the uſual way. The axillary glands were ſlightly affected, and the puſtule was ſmaller than what is commonly excited on the arm. On the eighth day, I took ſome matter from my hand and inoculated a young man, who has at this time the Vaccine diſeaſe in the moſt unequivocal manner.

I ſhall not enter into any argument on the propriety or impropriety of the term *ſpurious*. I have here uſed it as it has been generally accepted. I ſhall conclude with a hope that the Medical Gentlemen of this Empire, and others whoſe authority and example may have influence with the lower orders of ſociety, will cordially co-operate in adopting and ſupporting a practice which has for its object the total extinction of the Small-pox. The preſent age will then triumph in having vanquiſhed that foe to health, to beauty, and to life, while its name only will be known to poſterity.

If you think the above worthy a place in your Journal, you will oblige me by inſerting it.

I remain, &c.

*Burbage, Wilts, Jan.* 20, 1802. G. C. JENNER.

